# Inflammatory Signals shift from adipose to liver during high fat feeding and influence the development of steatohepatitis in mice

**DOI:** 10.1186/1476-9255-8-8

**Published:** 2011-03-16

**Authors:** Michaela C Stanton, Shu-Cheng Chen, James V Jackson, Alberto Rojas-Triana, David Kinsley, Long Cui, Jay S Fine, Scott Greenfeder, Loretta A Bober, Chung-Her Jenh

**Affiliations:** 1 Department of Cardiovascular and Metabolic Disease Research, Merck Research Laboratories (formerly Schering-Plough Research Institute), 2015 Galloping Hill Road, Kenilworth, NJ 07033, USA; 2 Department of Inflammation, Merck Research Laboratories (formerly Schering-Plough Research Institute), 2015 Galloping Hill Road, Kenilworth, NJ 07033, USA; 3 Current Address: Hoffmann-La Roche Inc., Nutley, NJ, USA

## Abstract

**Background:**

Obesity and inflammation are highly integrated processes in the pathogenesis of insulin resistance, diabetes, dyslipidemia, and non-alcoholic fatty liver disease. Molecular mechanisms underlying inflammatory events during high fat diet-induced obesity are poorly defined in mouse models of obesity. This work investigated gene activation signals integral to the temporal development of obesity.

**Methods:**

Gene expression analysis in multiple organs from obese mice was done with Taqman Low Density Array (TLDA) using a panel of 92 genes representing cell markers, cytokines, chemokines, metabolic, and activation genes. Mice were monitored for systemic changes characteristic of the disease, including hyperinsulinemia, body weight, and liver enzymes. Liver steatosis and fibrosis as well as cellular infiltrates in liver and adipose tissues were analyzed by histology and immunohistochemistry.

**Results:**

Obese C57BL/6 mice were fed with high fat and cholesterol diet (HFC) for 6, 16 and 26 weeks. Here we report that the mRNA levels of macrophage and inflammation associated genes were strongly upregulated at different time points in adipose tissues (6-16 weeks) and liver (16-26 weeks), after the start of HFC feeding. CD11b^+ ^and CD11c^+ ^macrophages highly infiltrated HFC liver at 16 and 26 weeks. We found clear evidence that signals for IL-1β, IL1RN, TNF-α and TGFβ-1 are present in both adipose and liver tissues and that these are linked to the development of inflammation and insulin resistance in the HFC-fed mice.

**Conclusions:**

Macrophage infiltration accompanied by severe inflammation and metabolic changes occurred in both adipose and liver tissues with a temporal shift in these signals depending upon the duration of HFC feeding. The evidences of gene expression profile, elevated serum alanine aminotransferase, and histological data support a progression towards nonalcoholic fatty liver disease and steatohepatitis in these HFC-fed mice within the time frame of 26 weeks.

## Background

Increased adiposity with the consequence of chronic low-grade inflammation and insulin resistance or type 2 diabetes has been linked to the development of nonalcoholic fatty liver disease (NAFLD). Currently, up to 30 percent of the general population is affected by NAFLD with 35 to 50 percent of obese adults also being diagnosed with nonalcoholic steatohepatitis (NASH). NAFLD has been described as the emerging clinical problem for the obese patient in the 21^st ^century [1]. The pathways that are active in promoting this disease process in the liver both in humans and in mouse models are poorly understood and are an active area of research.

There are a number of observations in the literature linking adiposity with inflammation and increased liver disease. Adipose tissue from obese people contains an increased number of CD68^+ ^macrophages with a pro-inflammatory phenotype [2]. In insulin-resistant patients with fatty liver disease, there is a significant upregulation of genes involved in fatty acid partitioning and binding proteins, monocyte recruitment and inflammation [3]. Obese mice demonstrate a significant increase in plasminogen activator in the fatty liver [4]. Likewise, the absence of CCR2 protects the liver against fat accumulation in the diet-induced obese mouse [5].

In the attempt to model the human disease process in rodents, researchers have used several versions of the Western diet and have found differences in severity of disease and times of disease onset depending upon the type of fats used for feeding. Mice fed diets high in trans fats combined with high fructose in the drinking water develop very aggressive liver disease within two months whereas mice fed only 20% of calories from high fat develop liver disease in nine months [6,7]. The genetic background of the rodent (C57BL/6 versus DBA/2) as well as cholesterol content of the diet and even the presence of endotoxin has been documented to strongly influence the development pattern of liver disease [8,9]. Zheng et al. [10] in our institution use a rodent model which incorporates a 45% fat diet with 0.12% cholesterol to reflect approximate percentages found in the Western diet. This rodent model has all the hallmarks of obesity, insulin resistance, and liver steatosis plus it offers the further advantage of proven use for the investigation of therapeutic drugs relevant to these diseases, such as ezetimibe [10].

As a prelude to the use of the model in other drug studies, we attempted to determine the molecular pathways that were activated in this mouse model of high fat and cholesterol (HFC) feeding as the syndrome progressed towards liver steatosis and fibrosis. We used a sensitive and high throughput technology, Taqman Low Density Array (TLDA) to study message expression profiling of 92 genes representing macrophage-associated, inflammation-related and metabolism-driven genes in various tissues, including the adipose tissues and liver at 6 weeks, midway at 16 weeks and at 26 weeks post-HFC feeding. We report here that there is an initial upregulation of genes in the epididymal adipose tissue that is accompanied by a relatively quiescent liver profile at 6 weeks post-HFC followed by a dramatic shift in emphasis away from the epididymal adipose tissue to liver tissue gene activation at 16 weeks and 26 weeks. Capturing changes in gene expression profiles from different organ systems as disease progression of the liver is actively occurring will allow valuable information on molecular mechanisms leading to NAFLD and NASH to be gathered in animal models of obesity and will lead to the identification of new therapeutic targets.

## Methods

### Animals and Diet

Six week old C57BL/6 male mice (Charles River Laboratories, Wilmington, MA) were housed in individual cages and kept at a temperature of 22°C and maintained on a 12:12 h light/dark cycle. Three separate cohorts of mice were used for these experiments so that evaluations could be performed at 6 weeks, 16 weeks and 26 weeks post-high fat feeding. Mice were fed a semi-purified diet containing high fat and cholesterol (45% Kcal from lard/soybean oil; 20% Kcal from protein; 35% Kcal from carbohydrate and 0.12% cholesterol by weight obtained from Research Diets (D0401280; New Brunswick, NJ) beginning at 7 weeks of age. Separate cohorts of age-matched normal animals were maintained on regular chow (Purina #5053) which provides 24.65% Kcal from protein; 62.14% Kcal from carbohydrate; and 13.2% Kcal from fat. The mineral and vitamin components were comparable between the two diets. C57BL/6 mice do not all gain weight on a uniform basis when fed this high fat diet. In order to minimize variability in our gene analysis results, mice were selected for their susceptibility to diet-induced obesity at day 21 following the start of high fat and cholesterol (HFC) feeding. Animals were considered to be diet-obese (DIO) if there was a seven gram body weight gain or greater after 21 days. In the cohorts of 150 mice started for each of these experiments, approximately 17% of mice fail this selection criterion on day 21 and are eliminated from further study. Body weight was followed throughout the course of the experiment. Total body fat was determined by use of a whole body magnetic resonance imager (EchoMR11200; Echo Medical Systems, Houston, TX).

The blood samples for analysis of insulin and glucose were taken from overnight-fasted animals in the morning at approximately 10 am. This measurement was done about three days prior to termination of the group. Glucose and insulin concentrations (in Table 1) are presented in International Units as mmol/l and pmol/l, respectively. Homeostatic model assessment (HOMA) values were calculated as an estimate of insulin sensitivity using the formula: fasting plasma glucose (mmol/l) × insulin (μU/ml) divided by 22.5. Higher values of HOMA indicate the presence of reduced insulin sensitivity in the animals [11]. The conversion of insulin concentration from International Units is 1 μU/ml = 6 pmol/l. This conversion factor is stated in the SI units table of the Journal of Diabetes Care.

**Table 1:** Assessment of serum metabolic parameters in diet-induced obese mice post-HFC initiation.

Parameter		6 weeks			16 weeks			26 weeks	
	**HFC**	**Chow**	**fold change**	**HFC**	**Chow**	**fold change**	**HFC**	**Chow**	**fold change**
	
Epididymal									
fat pad, %	5.6 (0.8)	2.8 (0.1)	2.0	2.6 (0.2)*	3.8 (0.3)	0.7	2.4 (0.3)**	4.2 (0.4)	0.6
Mesenteric									
fat pad, %	1.6 (0.1)*	0.9 (0.1)	1.8	2.1 (0.1)	1.6 (0.2)	1.3	1.9 (0.1)	1.9 (0.2)	1.0
Liver, %	3.6 (0.3)	3.7 (2)	1.0	7.3 (0.4)*	4.5 (0.4)	1.6	7.1 (0.4)*	4.6 (0.1)	1.5
ALT, U/ml	17 (2)	24 (3)	0.7	151 (24)*	31 (2)	4.9	76 (12)*	8 (1)	9.5
glucose, mmol/l	10.24 (0.25)	8.52 (0.25)	1.2	15.13 (0.54)	14.04 (0.46)	1.1	11.35 (0.28)	11.54 (0.34)	1.0
insulin, pmol/l	41.96 (2.81)*	28.85 (6.17)	1.5	436.49 (86.01)*	74.22 (9.97)	5.9	490.22 (55.19)*	278.26 (36.93)	1.8
HOMA	3.19 (0.24)*	1.92 (0.47)	1.7	47.83 (9.26)*	7.76 (1.14)	6.2	39.10 (3.35)*	24.12 (3.53)	1.6
adiponectin, μg/ml	6.7 (0.7)	6.0 (1)	1.1	12 (0.4)*	16 (0.8)	0.8	23 (3)	21 (2)	1.1
leptin, ng/ml	43 (10)*	0.5 (0.2)	86. 0	22 (6)*	4 (1)	5.5	46 (8)*	17 (3)	2.7
MCP-1, pg/ml	32 (2)*	26 (2)	1.2	36 (2)*	23 (2)	1.6	302 (26)*	134 (8)	2.3
IL-6, pg/ml	7 (1)	6 (1)	1.2	25 (6)*	12 (2)	2.1	33 (9)*	17 (5)	1.9
KC, pg/ml	32 (2)	21 (1)	1.5	67 (4)	44 (8)	1.5	98 (12)*	45 (4)	2.2
IL-10, pg/ml	15 (2)	27 (6)	0.6	124 (43)	47 (6)	2.6	45 (17)*	17 (6)	2.6
serum amyloid A									
μg/ml	1.1 (0.02)	0.5 (0.2)	2.2	1.6 (0.7)	0.81 (0.05)	2.0	1.85 (0.2)*	0.43 (0.06)	4.3

Assessment was done at the termination point of 6, 16 or 26 weeks post-HFC initiation.

Values are **means (sem)**, n = 14-20 per group. * confidence interval = 95%; ** confidence interval = 99%.

The fold change is calculated as the level in HFC group divided by the level obtained from the Chow group.

The levels of GM-CSF, TNF-α, IL-12p70 and IL-1β were below detection limit of the assays.

Blood samples for lipid profile, cytokine analysis and liver enzymes were taken on the day of termination from non-fasted animals at approximately the same time. All studies were carried out in our vivarium in accordance with the Guide for the Care and Use of Laboratory Animals of the National Institutes of Health and the Animal Welfare Act under the supervision of our institutional Animal Care and Use Committee.

### Serum Cytokines and Other Mediators

Serum was evaluated for GM-CSF, insulin, leptin, MCP-1, IL-6, TNF-α, IL-10, IL12p70, IL-1β, KC (Meso Scale Discovery, Gaithersburg, MD); serum amyloid A (Life Diagnostics, West Chester, PA); alanine aminotransferase (ALT) (Catachem, Bridgeport, CA) and adiponectin (R&D Diagnostics, Minneapolis, MN). Data from cytokine and mediator evaluation is reported as the mean (sem) of the group. All statistical analysis was performed by Mann-Whitney U test using GraphPad Instat version 3.06 for Windows XP (GraphPad Software, San Diego, CA).

### Histology and immunohistochemistry (IHC)

5 μm paraffin sections were stained by either hematoxylin and eosin (H&E) or Masson trichrome stain [12]. For IHC and oil red O staining, frozen liver or adipose tissues embedded in OCT were cut at 5 (IHC) or 10 μm (oil red O) and freshly frozen in -80°C freezer until use. After fixation with acetone, tissue sections were incubated with anti-CD11b (BD Bioscience), anti-CD11c (Endogen), anti-IL-1β (R&D) or anti-F4/80 (Serotec) for 1 h at room temperature followed by incubation with either biotinylated rabbit anti-rat or donkey anti-goat antibodies. Selective binding was visualized by the enzymatic reaction of an alkaline phosphatase (ABC kit, Vector) with its substrate, permanent red (Dako). Hematoxylin was used for counterstaining. Oil red O staining was carried out as described [13].

### RNA isolation and quantitative RT-PCR

#### Tissue collection and homogenization

Approximately 300-500 μl of blood from each mouse was collected and added to a PAXgene blood RNA tube containing ~1.3 ml of a proprietary reagent developed by PreAnalytiX. Pancreas was isolated using a method adapted from Mullin et al. [14]. Remaining tissues (mesenteric lymph nodes, mesenteric fat pad, epididymal fat pad, spleen, liver and gastrocnemius muscle) were excised and flash frozen in liquid nitrogen.

A TissueLyser (Qiagen, Valencia, CA) was used to homogenize and disrupt collected tissues in preparation for total RNA extraction. A sterile 5 mm stainless steel bead and 1 ml QIAzol lysis reagent (for epididymal and mesenteric fat pads), 350 μl buffer RLT (for mesenteric lymph nodes) or 2 ml buffer RLT (for liver, spleen and gastrocnemius muscle) was added to each 2 ml eppendorf tube containing the frozen tissue piece. Tissues were then agitated at 30 Hz for 2 × 2 minutes as per the recommendations of the Qiagen TissueLyser handbook. A handheld TissueMiser (Thermo-Fisher Scientific) was used to homogenize and disrupt the pancreas tissues.

#### RNA isolation and cDNA synthesis

Total RNA isolation from all tissues was performed according to manufacturer's protocol (Qiagen, Valencia, CA). Optional on column DNase digestion was performed on all tissues. Total RNA from blood was isolated on the day it was collected using PAXgene Blood RNA kit. All isolated total RNA was stored at -80°C until further use. RNA was quantified using the NanoDrop^® ^ND-1000 spectrophotometer (Agilent Technologies, Santa Clara, CA). RNA quality was assessed by running a 500-2000 ng sample on a MOPS buffered formaldehyde gel. First strand cDNA synthesis was performed using the Applied Biosystems High Capacity cDNA Reverse Transcription kit (Applied Biosystems, Foster City, CA) according to manufacturer's instructions. To ensure equal loading of all samples on the TLDA card, cDNA was quantified against an 18S standard curve prepared using human universal reference total RNA purchased from Clontech (BD Biosciences Clontech, Heidelberg, Germany).

#### Taqman Low Density Array

Quantitative real-time PCR utilized custom made TaqMan^® ^Low Density Array (TLDA) from Applied Biosystems and followed the manufacturer's instructions. Thermal cycling was performed using an ABI Prism 7900HT Sequence Detection System. 100 ng cDNA in 100 μl of Applied Biosystems 1X Universal PCR Master mix was loaded onto each port of the TLDA plates. Data was analyzed using SDS v2.2 software. The Ct value of each gene is normalized to 18S to obtain ∆Ct. Relative quantitation or fold changes in gene expression were determined using the formula **2 **^-∆ ∆ **Ct**^, where ∆∆Ct = average ∆Ct of all HFC-fed samples - average ∆Ct of all chow-fed samples. Statistical significance was determined by two-tailed Welch t test using either GraphPad Prism 4 or Microsoft Excel 2003, where *P *< 0.05 (*), *P *< 0.01 (**), and *P *< 0.001 (***). Unmarked data points are not significant. The numbers of mice in each group are as follows: 7 Chow-fed and 15 HFC-fed mice at 6 weeks; 8 Chow-fed and 10 HFC-fed mice at 16 weeks; and 10 Chow-fed and 12 HFC-fed mice at 26 weeks.

## Results

**Figure 1:**
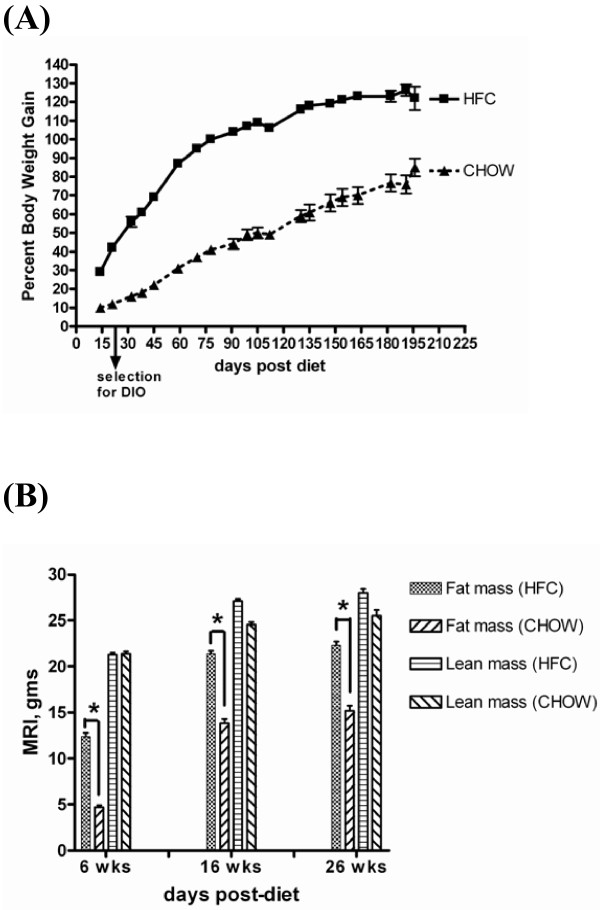
**Percent body weight gain and fat mass increase in HFC-fed mice over time**. A: Percent body weight gain over time. All time points plotted are *P *< 0.01 for 45% high fat + 0.12% cholesterol (HFC) vs. chow-diet (CHOW), Mann-Whitney U test. Animals selected at day 21 for increased body weight (DIO; diet-induced obesity). B: Body Density Parameters determined by MRI Analysis. *P < 0.0001 for fat mass of HFC vs. CHOW, Mann-Whitney U test.

To qualify our animal model as described previously by Zheng et al. [10] we have characterized the animals by tracking their body weight changes and the levels of serum mediators and cytokines throughout the time course. The percent body weight increased progressively in the HFC-fed mice over the 6 to 16 week study period and was maximal at 26 weeks post-HFC (Figure 1A). This body weight increase was accompanied by an increase in fat mass (gms) determined by MRI (Figure 1B). There was no effect of diet treatment on lean body mass. The HOMA index (Table 1) indicates that the high fat fed mice developed a significant degree of insulin resistance at the time points measured for this experiment. The epididymal fat pad measured at 6 weeks was the organ most strikingly affected when compared to the chow-fed animals. However, as the experiment progressed to 16 and 26 weeks, the epididymal fat pad weight as a percent of body weight actually decreased (Table 1). The liver weight (expressed as a percent of body weight) of the 6-week HFC-fed mice was unchanged from chow-fed controls; however, the liver weight of 16- and 26-week HFC-fed mice showed a continuous increase relative to the chow-fed mice. This increase in liver weight at 16 and 26 weeks was accompanied by an increase in the serum levels of alanine aminotransferase (ALT), indicative of progressive liver damage (Table 1).

We measured a variety of serum cytokines and mediators from these animals at the observation points. We found that there was a large degree of variability in these animals despite pre-selection for diet-induced obesity (DIO). We routinely kept the animals on a HFC diet for 3 weeks prior to entrance into the experimental cohorts to ensure that all animals chosen had at least a 30% increase in body weight when compared to chow-fed mice. Of the adipokines measured, serum leptin levels continually increased over time (Table 1). Adiponectin decreased only at 16 weeks of HFC feeding. Of the chemokines tested, MCP-1 (CCL2) was elevated throughout the observation periods in the HFC-fed mice; KC levels although higher than those of the chow-fed mice were not significantly elevated until 26 weeks post-HFC. Of the pro-inflammatory cytokines measured, IL-6 showed a modest increase at 16 and 26 weeks post-HFC. We did not obtain appreciable increases in circulating levels of GM-CSF, TNF-α, IL-12p70 and IL-1β in these HFC-mice. Serum amyloid A (SAA) levels were variable at 6 and 16 weeks post-HFC but were significantly elevated in the HFC-fed mice at 26 weeks post-HFC. IL-10 levels were increased in the serum of the HFC-fed mice at 16 weeks but were highly variable. At 26 weeks, IL-10 levels were more consistently elevated over the chow-fed controls. These measurements over the course of HFC feeding demonstrated that there was an inflammatory milieu in these mice.

### Histological analysis reveals hepatic steatosis and inflammation in HFC-fed mice

**Figure 2:**
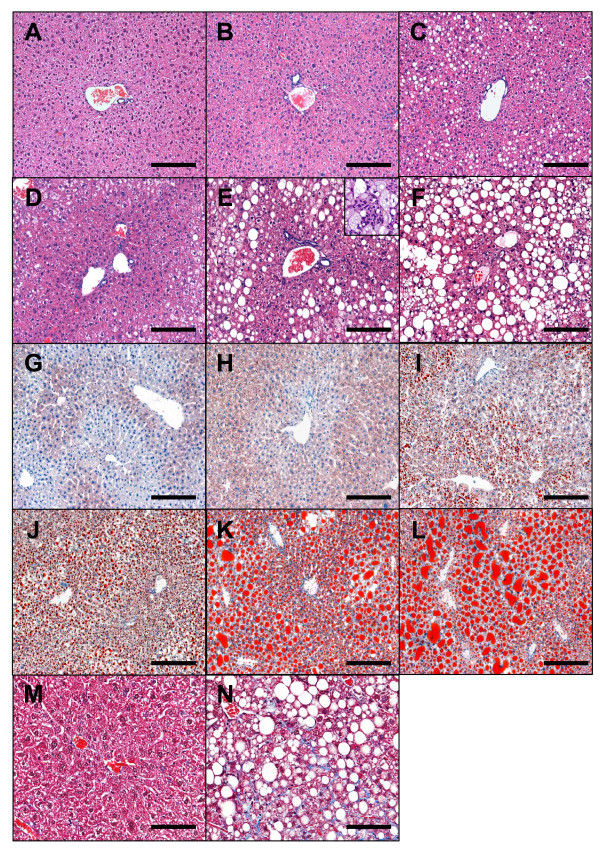
**Steatosis, inflammation and fibrosis in livers of HFC-fed mice**. Liver sections from 6 (A, D, G, J), 16 (B, E, H, K, M, N) and 26 (C, F, I, L) weeks of chow (A-C, G-I, M) and HFC (D-F, J-L, N) fed mice were analyzed histologically. **A-F**, H&E stain. Cellular infiltrates are readily seen throughout 16 and 26 weeks of HFC livers and is illustrated in the insert of E. **G-L**, Oil red O stain. Increased focal fibrosis as demonstrated by trichrome stain was found in livers of some HFC-fed mice at 16 weeks (N) or later as compared to 16 week chow-fed liver (M). A-L bar = 0.15 mm. M&N, bar = 0.075 mm.

Histological examination with both H&E and oil red O staining of liver sections from HFC-fed mice demonstrated a progressive development of steatosis coupled with inflammation as shown in Figure 2. No macrovesicular steatosis was observed in livers from chow-fed mice at 6 and 16 weeks (Figure 2, A-B for H&E and 2G-H for oil red O). Low grade macrovesicular steatosis was observed in the chow-fed group only at week 26 (Figure 2C for H&E and 2I for oil red O). In contrast to the chow-fed group, macrovesicular steatosis was observed in HFC liver as early as 6 weeks after exposure to HFC diet. At this time point, the fat droplets were distributed in zone 2 and 3 with the majority in the intermediate zone (zone 2) between portal and central veins as shown on H&E stained section (Figure 2D) and this observation was further confirmed with oil red O staining (Figure 2J). No cytoplasmic foamy changes were found at this time. The number and the size of fat droplets were dramatically increased by week 16 and 26 as evident from sections stained with oil-red O (Figure 2, E-F for H&E and 2K-L for oil red O). In addition to steatosis, signs of inflammation including infiltration of inflammatory cells (see insert of Figure 2E) and focal fibrosis, revealed by trichrome stain (Figure 2M and 2N) were readily observed in the HFC liver at 16-26 weeks post-HFC.

### Gene expression profiling reveals profound inflammatory gene regulation specifically in adipose and liver tissues of HFC-fed mice

To study the molecular mechanisms and pathways underlying chronic inflammation and insulin resistance, we utilized a custom-designed gene card to perform Taqman Low Density Array (TLDA) with multiple tissues taken from HFC- and chow-fed mice. We used previous comparisons to validate the results from TLDA by conventional quantitative real-time RT-PCR which then allowed us to choose TLDA as a high throughput assay for multiple gene expression profiling throughout this study. The gene card contains 92 unique genes chosen from their known functions associated with macrophages, adipokines, cytokines, chemokines, insulin signalling, endoplasmic reticulum stress, and glucose, lipid and energy metabolism (see Additional File 1 for details). The overall gene expression profiling reveals profound gene regulation in epididymal adipose tissue, mesenteric adipose tissue and liver (summarized in Additional File 2). There was either minor or no change of these genes in blood cells, muscle, pancreas, spleen and lymph nodes, based mostly on the results from pooled RNA samples (see Additional File 3). Our gene expression profiles in adipose and liver tissues established that there is a definitive presence of macrophage infiltration and inflammatory signals that is induced by obesity in HFC-fed mice. Here, we describe differential regulation of several groups of important genes involved in chronic inflammation and insulin resistance in adipose (epididymal and mesenteric fat pads) and liver tissues.

### mRNA levels of genes involved in macrophage recruitment are strongly upregulated early in adipose tissues and progressively switched to liver of HFC- fed mice

**Figure 3:**
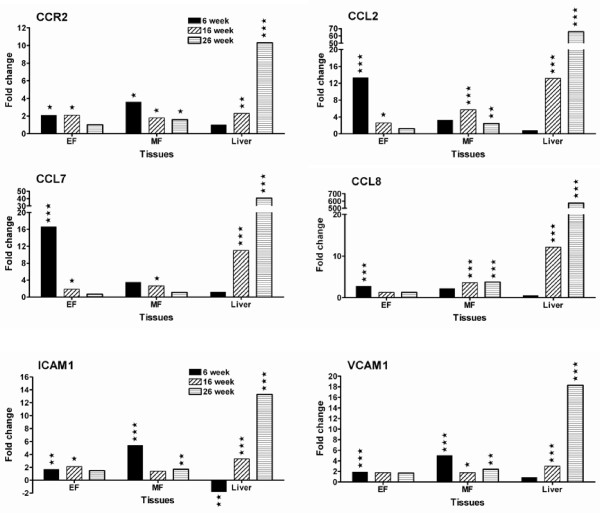
**Genes involved in macrophage recruitment are differentially upregulated in adipose and liver tissues of HFC-fed mice**. EF stands for epididymal fat pad and MF for mesenteric fat pad. Data are presented as fold change of mRNA levels in HFC group vs. chow group. Statistical significance was determined by two-tailed Welch t test where *P *< 0.05 (*), *P *< 0.01 (**), and *P *< 0.001 (***) (details in Methods).

mRNA levels of genes involved in macrophage recruitment including inflammatory chemokines (CCL2, CCL7, CCL8), chemokine receptor (CCR2) and adhesion molecules (ICAM1, VCAM1), were upregulated in epididymal (EF) adipose tissues at 6 weeks of HFC feeding (Figure 3). In contrast, in mesenteric (MF) adipose tissue at this time period, only the mRNA levels of genes coding for CCR2, ICAM1, VCAM1 were upregulated but not those of the chemokines. This differential upregulation may provide the early inflammatory signal for recruiting circulating monocytes into the adipose tissues of different areas. At this time point, there was no significant change in expression of these genes in liver.

The strong upregulation of mRNA levels of these genes in adipose tissues at 6 weeks was mostly decreased when the duration of HFC feeding increased to 16 weeks and 26 weeks. The dramatic decrease of relative mRNA level (fold change) at 16 weeks resulted from a decrease of mRNA levels in the HFC group and a concomitant increase of mRNA levels in the chow group. Intriguingly, mRNA levels of these genes were highly upregulated in liver at 16 weeks and even further increased at 26 weeks of HFC feeding (Figure 3). This is the first finding of significant gene regulation in the liver of these obese mice.

### HFC diet induces macrophage infiltration and accumulation in adipose and liver tissues

**Figure 4:**
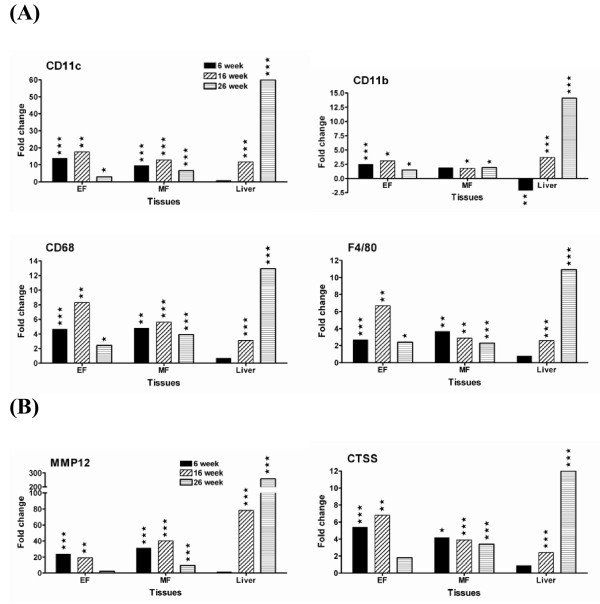
**Strong upregulation of mRNA levels of macrophage markers and proteases provides a direct evidence for macrophage infiltration**. (A) macrophage markers and (B) proteases. EF stands for epididymal fat pad and MF for mesenteric fat pad. Data are presented as fold change of mRNA levels in HFC group vs. chow group. Statistical significance was determined by two-tailed Welch t test where *P *< 0.05 (*), *P *< 0.01 (**), and *P *< 0.001 (***) (details in Methods).

To investigate macrophage infiltration and accumulation following exposure to HFC diet, gene expression profiles of several macrophage markers and proteases were evaluated. As shown in Figure 4, mRNA levels of four macrophage markers CD11c, CD11b, CD68 and F4/80, were highly upregulated in HFC adipose tissue at all time points analyzed as compared to chow-fed mice and peaked at 16 weeks of HFC feeding (Figure 4A). Another macrophage marker CD83 was upregulated in a similar manner (See Additional File 2). Two proteases (MMP12 and CTSS) known to be highly expressed in macrophages also had a similar gene expression profile as those macrophage markers (Figure 4B). Again, significant upregulation of these macrophage markers in liver was delayed until 16 weeks of HFC feeding.

**Figure 5:**
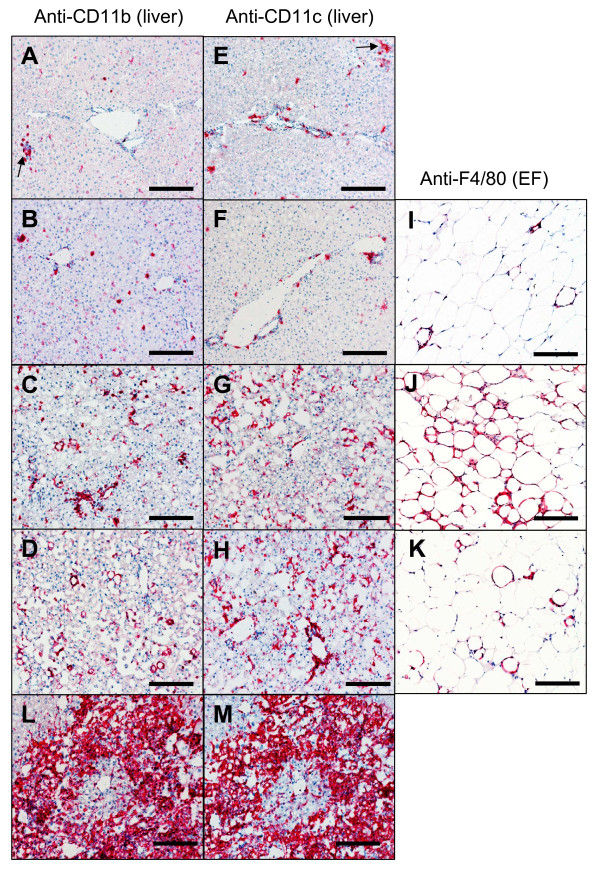
**Macrophage infiltration in HFC-fed liver and adipose tissues**. Liver (A-H, L&M) and epididymal fat (I-K) tissues from 6 week chow-fed (A, E), 6 week HFC-fed (B, F, I), 16 week HFC-fed (C, G, J, L, M) and 26 week HFC-fed (D, H, K) mice were analyzed with immunohistochemistry using anti-CD11b (A-D), anti-CD11c (E-H) and anti-F4/80 (I-K). L&M are adjacent sections incubated with either anti-CD11b (L) or anti-CD11c (M) demonstrating similar patterns of cellular infiltrates in the same area of the sections. Arrows in A&E point to groups of aggregates associated with EMH. bar = 0.15 mm.

To confirm increased macrophage accumulation in the liver we performed IHC with anti-CD11b and anti-CD11c antibodies (Figure 5). Occasionally, small groups of CD11b^+ ^or CD11c^+ ^aggregates were observed among the groups of extramedullary hematopoietic (EMH) cells (Figure 5A and 5B). Consistent with findings by RT-PCR, no significant increase of CD11b^+ ^or CD11c^+ ^cells were found in livers from chow-fed groups at all time points (data of later time points not shown) or at 6 weeks post-HFC as compared to chow controls (Figure 5). However, at 16 and 26 weeks post-HFC, a significant increase in inflammatory cell numbers was found in the liver sections of the HFC mice. In addition to the increased numbers of cells at these time points, these cells also appeared to be enlarged and demonstrated a morphology suggesting an activated state, which was consistent with the upregulation of CD83 mRNA. Macrophage infiltration into adipose tissues was also investigated throughout the same time course. Consistent to TLDA data, in the epididymal fat (EF) macrophage infiltrates peaked at week 16 and declined at week 26 post-HFC (Figure 5I-K). Occasionally, focal massive infiltrates of CD11b^+ ^or CD11c^+ ^cells were also observed in both 16- and 26-week HFC livers (Figure 5L and 5M). These two populations of cells appear to co-exist in the same area as demonstrated by the use of adjacent sections.

### mRNA levels of pro-inflammatory cytokine genes are differentially upregulated in both adipose and liver tissues of HFC-fed mice

**Figure 6:**
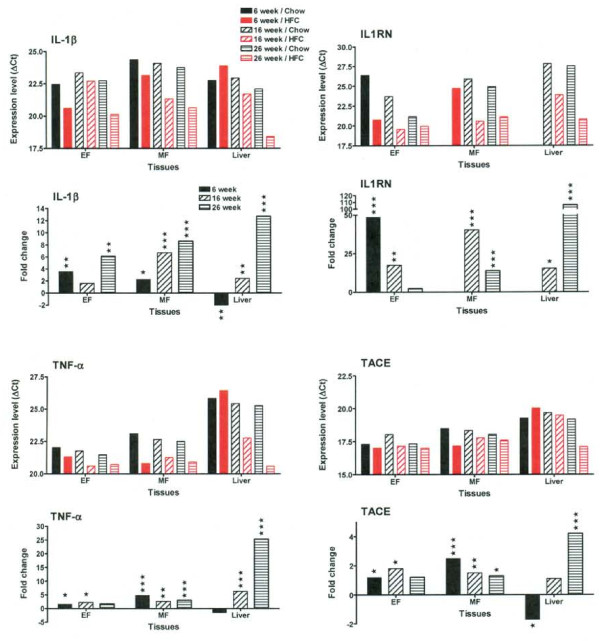
**IL-1β, IL1RN, TNF-α and TACE genes are differentially upregulated in both adipose and liver tissues of HFC-fed mice**. Expression levels of IL-1β, IL1RN, TNF-α and TACE genes from chow (in black) and HFC (in red) fed mice at each time point are presented as average ∆Ct of all animals in each group (details in Methods). The smaller ∆Ct value indicates the higher expression level. EF stands for epididymal fat pad and MF for mesenteric fat pad. The MF sample of 6 week/Chow and the liver samples of 6 week/Chow and 6 week/HFC had no signal for IL1RN because of very low expression level. In addition, the fold change of mRNA levels in HFC group vs. chow group is also presented below the expression level panel for each gene. Statistical significance was determined by two-tailed Welch t test where *P *< 0.05 (*), *P *< 0.01 (**), and *P *< 0.001 (***).

**Figure 7:**
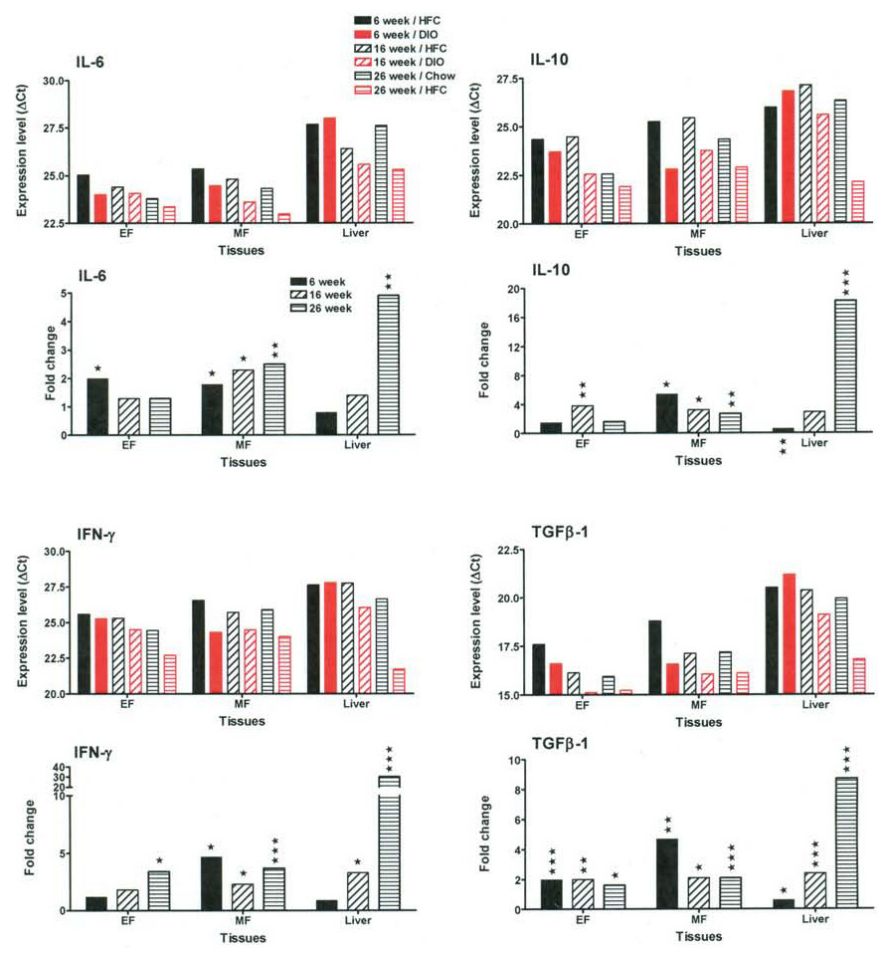
**IL-6, IL-10, IFN-γ and TGFβ-1 genes are differentially upregulated in both adipose and liver tissues of HFC-fed mice**. Expression levels of IL-6, IL-10, IFN-γ and TGFβ-1 from chow (in black) and HFC (in red) fed mice at each time point are presented as average ∆Ct of all animals in each group. The smaller ∆Ct value indicates the higher expression level. EF stands for epididymal fat pad and MF for mesenteric fat pad. In addition, the fold change of mRNA levels in HFC group vs. chow group is also presented below the expression level panel for each gene. Statistical significance was determined by two-tailed Welch t test where *P *< 0.05 (*), *P *< 0.01 (**), and *P *< 0.001 (***).

A complex regulation of pro-inflammatory cytokine genes was observed at different time points in both adipose and liver tissues, underlying both disease-promoting and compensatory mechanisms (Figure 6 and 7). As an example, we determined that the mRNA level of IL-1β increased throughout the time course in both adipose tissues (EF and MF), as shown by both decrease in ∆Ct (increase in expression level) and increase in fold change (Figure 6). However, analysis of IL-1 receptor antagonist (IL1RN) showed that although there was a dramatic increase at 6 weeks of HFC feeding, this was followed by a substantial decrease in the expression level of IL1RN at 16 weeks and 26 weeks of HFC feeding. In contrast, IL-18 was not significantly regulated in the HFC-fed mice (See Additional File 2). In addition, the relative mRNA levels of TNF-α, TACE (Figure 6) and TGFβ-1 (Figure 7) were upregulated throughout the time course in both adipose tissues. The relative mRNA levels of IL-6, IL-10 and IFN-γ were consistently elevated in mesenteric (MF) adipose tissue, rather than in epididymal (EF) adipose tissue (Figure 7).

**Figure 8:**
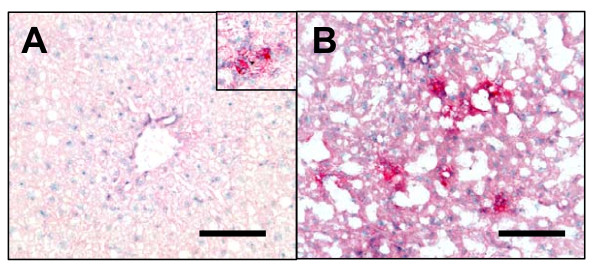
**Increased number of IL-1β**^**+ **^**cells in the liver of HFC-fed mice**. Immunohistochemistry of anti-IL-1β demonstrated increased number of IL-1β^+ ^cells in HFC-fed liver at 16 weeks (*B*) as compared to 16 week chow-fed liver (*A*). Occasionally, small groups of IL-1β^+ ^aggregates were also detected in chow-fed livers at all time points (insert of *A*, 6 week chow-fed). bar = 0.075 mm.

In the liver, mRNA levels of IL-1β, IL1RN, TNF-α, IFN-γ and TGFβ-1 were highly upregulated at 16 weeks of HFC feeding and further increased at 26 weeks (Figure 6 and 7), suggesting the presence of severe inflammation. To confirm an elevation at the protein level, we examined the expression of IL-1β in liver by IHC (Figure 8). In general, there was no significant IL-1β expression in chow-fed livers, except for occasional small groups of aggregates, as shown in the insert of Figure 8A. Consistent with its mRNA profile, the numbers of IL-1β^+ ^cells increased with time in HFC-fed animals (Figure 8B) indicating the relevance of IL-1β to liver inflammation. No significant change of IL-1β expression was observed throughout the time course in the livers of the chow-fed animals.

## Discussion

Our work on gene activation in the HFC-fed mouse model is an attempt to accurately predict the sites of drug intervention and to possibly discover new targets in the mechanisms leading up to the induction of overt NASH disease. This paper describes a kinetic study for the direct temporal and spatial comparison of gene activation in a variety of organs as compared to age-matched chow-fed controls. First, a time dependent, diet-induced development of liver histopathology reminiscent of NASH, including steatosis, fibrosis and inflammatory infiltrates, was observed in the animal model used in our study of gene expression. Analysis of temporal inflammatory gene expression in multiple organs was carried out by TLDA. The epididymal adipose, mesenteric adipose and liver tissues show definitive time-dependent gene activation. In particular, those genes representing inflammatory mediators change dramatically over time. Gene activation to a various degree was also observed in other organ systems such as the blood cells, spleen, lymph nodes, gastrocnemius muscle, or pancreas but these gene patterns do not appear in any discernible pattern that would impact the induction of liver disease.

We report here that gene activation occurs early in the epididymal adipose with the peak activation at 6 weeks post-HFC. This activity in this particular adipose tissue is followed by a definitive increase in inflammatory signals in the liver at 16 weeks and 26 weeks post-HFC feeding. On the contrary, activated genes were downregulated in the epididymal fat pad at these later time points. This switch in activation profile from epididymal adipose tissue to liver as determined by quantitative RT-PCR was corroborated by the finding that there were significant increases in CD11b^+ ^or CD11c^+ ^macrophages in the liver at 16 weeks and 26 weeks post-HFC as well as by an increased accumulation of fatty droplets in the liver. These "activated macrophages" were not seen in the 6 week HFC livers when evaluated versus age-matched chow-fed control mice nor were there as many fatty droplets at this early time point. This increase in cellularity in the liver also appears in human disease. Genes involved in monocyte/macrophage recruitment are over-expressed in the livers of insulin-resistant human patients [3] and it is well-established that macrophages will accumulate both in adipose and liver under the influence of inflammatory signals [15,16]. Furthermore, the reduction in gene activation observed in the epididymal adipose tissues at the later time point was accompanied by a decreased number of macrophages in this fat pad at 26 weeks post-HFC. These data suggest that a trigger for induction of inflammation was first set off in the adipose tissue and then sent out to other organ systems as fat feeding continued over time. This adipose-initiated signal sets up a process which results in overt liver disease by 26 weeks.

TLDA results of the mesenteric adipose tissue from HFC-fed mice indicate that inflammatory mediators and cell activation signals are also induced in this fat depot. This adipose tissue, however, does not appear to shift as dramatically in gene activation profile as that of the epididymal fat pad. This continual generation of cytokine and mediator gene signals found within the mesenteric fat amplifies the shift toward enhanced gene activation of the liver in these HFC-fed mice. It has been reported that the omental fat pad in obese humans can serve as a steady-state generator of inflammatory mediators which will then impact the development of disease [17].

Within the panel of genes included in our analysis, which ranges from metabolic to cellular markers to inflammatory mediators, IL-1β is identified as one of the most relevant inflammatory mediator as the disease induction process shifts from the inflamed epididymal adipose tissue to the liver. This enhanced IL-1 signal is relevant to current research findings in human metabolic disease. In obese patients, mRNA levels of IL-1 receptor antagonist (IL-1Ra) and IL-1 receptor type 1(IL-1R1) are markedly upregulated in white adipose tissue. In addition, IL-1β gene expression was selectively increased in the visceral fat (but not subcutaneous fat) from obese subjects [18]. In patients with type 2 diabetes, the use of Anakinra (IL-1Ra) improved glycemia and beta-cell secretory function and reduced markers of systemic inflammation (CRP, IL-6) [19]. Recent positive clinical results from a small trial with high affinity anti-human IL-1β (XOMA 052) also support targeting IL-1β mediated inflammatory damage to pancreatic beta cells as a potential therapeutic approach for type 2 diabetes [20]. The use of TLDA technology applied to this mouse model can reveal further activated or dysregulated gene signals that will be relevant to human disease.

Excessive pro-inflammatory stimulation enhances the development of steatosis and NASH. In patients with acute and chronic liver diseases, production of IL-1 alters as the liver disease shifts in intensity from acute to chronic cirrhosis [21]. At present, it appears that a certain mix of cytokines plus a genetic predisposition to hepatic immune defects may be needed to facilitate progression of NASH to cirrhosis [22]. Our work demonstrates that there is very strong and relevant cross-talk between organ systems of the adipose tissue and the liver in our HFC mouse model which is also dependent upon the genotype of the mouse [23]. Use of the TLDA technology to track gene changes over a time line will help to unravel some of this immune conversation and to identify whether new therapeutics can make these gene signals quiescent.

## Conclusions

In the present study, we utilized the mouse model with a diet containing 45% fat and 0.12% cholesterol (as found in the Western diet) to investigate the molecular signals that trigger insulin resistance, hyperinsulinemia and progression to liver steatosis and fibrosis in the diet-induced obesity (DIO) mouse model. We used a sensitive and high throughput technology, Taqman Low Density Array (TLDA), to study gene expression profiling of 92 important genes representing macrophage-associated, inflammation-related and metabolism-driven genes in multiple tissues at 6 weeks, 16 weeks and 26 weeks post high fat and cholesterol (HFC) feeding. We demonstrate from our analysis of the gene activation profiles that macrophage infiltration accompanied by severe inflammation and metabolic changes occurs in both adipose and liver tissues with a temporal shift in the levels of these signals depending upon the duration of HFC feeding. This gene activation initiates early at 6 weeks post-HFC feeding in epididymal adipose tissues. Activation signals then switch to the liver at 16 and 26 weeks post-HFC feeding. These findings of time dependent development of steatosis in the liver are supported by immunohistochemistry. Taken together, the evidences of gene expression profile, elevated serum alanine aminotransferase, increased liver to body weight ratio, and histological data support a progression driven by diet-induced inflammation towards nonalcoholic fatty liver disease and even nonalcoholic steatohepatitis in these HFC-fed mice within the time frame of 26 weeks.

## Abbreviations

TLDA: taqman low density array; HFC: high fat and cholesterol diet; IL-1β: interleukin-1β; IL1RN: interleukin 1 receptor antagonist; TNF-α: tumor necrosis factor-α; TGFβ-1: transforming growth factor β-1; NAFLD: nonalcoholic fatty liver disease; NASH: nonalcoholic steatohepatitis; GM-CSF: granulocyte macrophage-colony stimulating factor; MCP-1: monocyte chemoattractant protein-1; KC: keratinocyte chemoattractant; ALT: alanine aminotransferase; CCL2: chemokine (C-C motif) ligand 2; CCR2: chemokine (C-C motif) receptor 2; ICAM1: intercellular adhesion molecule 1; VCAM1: vascular cell adhesion molecule 1; MMP12: matrix metalloproteinase 12; CTSS: cathepsin S; TACE: TNF-α converting enzyme; IFN-γ: interferon-γ; SAA: serum amyloid A.

## Competing interests

The authors declare that they have no competing interests.

## Authors' contributions

MCS isolated RNA from all tissues, carried out Taqman Low Density Array and data analyses. SCC designed the histologic and immunohistochemical studies, analyzed the data, drafted part of the manuscript and prepared the histology and IHC figures. ART and JVJ carried out mouse models and evaluated serum cytokines and other mediators. DK carried out the histology and IHC works. LC performed in situ ductal perfusion into the pancreas and harvested multiple tissues from all mice. JSF participated in the design of the study and selection of the gene list. SG participated in the design of the study, data discussion and supported the preparation of the manuscript. LAB supervised mouse models, participated in the design of the study, analyzed serum data and drafted part of the manuscript. CHJ designed the gene list, coordinated the study, performed statistical analyses and wrote the manuscript. All authors read and approved the final manuscript.

## Supplementary Material

Additional file 1 **Table S1**. The table shows a list of 92 genes designed on the gene card for Taqman Low Density Array. Table S1. The gene panel for gene expression study by Taqman Low Density Array Click here for file

Additional file 2 **Table S2, S3, and S4**. High fat and cholesterol diet (HFC) induced gene regulation in epididymal adipose tissues, mesenteric adipose tissues and liver of C57BL/6 mice. These tables contain the gene expression profile of all genes in this study. Table S2. The gene expression profile in **epididymal adipose tissues **of HFC-fed mice Table S3. The gene expression profile in **mesenteric adipose tissues **of HFC-fed mice. Table S4. The gene expression profile in **liver **of HFC-fed mice Click here for file

Additional file 3 **Table S5 and S6**. High fat and cholesterol diet (HFC) induced gene regulation in blood cell, muscle, spleen, lymph node and pancreas tissues of C57BL/6 mice. These tables contain the gene expression profile of all genes in this study, mostly from pooled RNAs without statistical analyses. Table S5. The gene expression profile in blood cells, muscle and spleen tissues. Table S6. The gene expression profile in lymph node and pancreas tissues Click here for file
