# Supplementary material for: Inflammatory Signals shift from adipose to liver during high fat feeding and influence the development of steatohepatitis in mice
Source: J Inflamm (Lond). 2011 Mar 16;8:8. doi: 10.1186/1476-9255-8-8 (PMC3070617; doi:10.1186/1476-9255-8-8)
Supplement: Additional file 1 — Table S1. The table shows a list of 92 genes designed on the gene card for Taqman Low Density Array. Table S1. The gene panel for gene expression study by Taqman Low Density Array [file 1476-9255-8-8-S1.DOC]

**Additional File 1**

Table S1 - The gene panel for gene expression study by Taqman Low Density Array
