# Supplementary material for: Inflammatory Signals shift from adipose to liver during high fat feeding and influence the development of steatohepatitis in mice
Source: J Inflamm (Lond). 2011 Mar 16;8:8. doi: 10.1186/1476-9255-8-8 (PMC3070617; doi:10.1186/1476-9255-8-8)
Supplement: Additional file 2 — Table S2, S3, and S4. High fat and cholesterol diet (HFC) induced gene regulation in epididymal adipose tissues, mesenteric adipose tissues and liver of C57BL/6 mice. These tables contain the gene expression profile of all genes in this study. Table S2. The gene expression profile in epididymal adipose tissues of HFC-fed mice Table S3. The gene expression profile in mesenteric adipose tissues of HFC-fed mice. Table S4. The gene expression profile in liver of HFC-fed mice [file 1476-9255-8-8-S2.DOC]

**Additional File 2**

Table S2. The gene expression profile in **epididymal adipose tissues** of HFC-fed mice

High fat and cholesterol diet (HFC) induced gene regulation in **epididymal adipose tissues** of C57BL/6 mice.

The relative mRNA level of each gene (indicated by fold change) was determined by calibrating against its respective chow-fed group at each time point. The blank indicates genes with no signal.

Table S3. The gene expression profile in **mesenteric adipose tissues** of HFC-fed mice

High fat and cholesterol diet (HFC) induced gene regulation in **mesenteric adipose tissues** of C57BL/6 mice.

The relative mRNA level of each gene (indicated by fold change) was determined by calibrating against its respective chow-fed group at each time point. The blank indicates genes with no signal.

Table S4. The gene expression profile in **liver** of HFC-fed mice

High fat and cholesterol diet (HFC) induced gene regulation in **liver** of C57BL/6 mice.

The relative mRNA level of each gene (indicated by fold change) was determined by calibrating against its respective chow-fed group at each time point. The blank indicates genes with no signal.
