# Supplementary material for: Inflammatory Signals shift from adipose to liver during high fat feeding and influence the development of steatohepatitis in mice
Source: J Inflamm (Lond). 2011 Mar 16;8:8. doi: 10.1186/1476-9255-8-8 (PMC3070617; doi:10.1186/1476-9255-8-8)
Supplement: Additional file 3 — Table S5 and S6. High fat and cholesterol diet (HFC) induced gene regulation in blood cell, muscle, spleen, lymph node and pancreas tissues of C57BL/6 mice. These tables contain the gene expression profile of all genes in this study, mostly from pooled RNAs without statistical analyses. Table S5. The gene expression profile in blood cells, muscle and spleen tissues. Table S6. The gene expression profile in lymph node and pancreas tissues [file 1476-9255-8-8-S3.DOC]

**Additional File 3**

Table S5. The gene expression profile in blood cells, muscle and spleen tissues

The gene expression profile of pooled RNAs in blood cells, muscle and spleen tissues of HFC-fed mice.

The relative mRNA level of each gene (indicated by fold change) was determined by using pooled RNAs from HFC-fed group calibrated against its respective chow-fed group at each time point without statistical analyses. The blank indicates genes with no signal or low copy.

Table S6. The gene expression profile in lymph node and pancreas tissues

The gene expression profile of pooled RNAs in lymph nodes and individual RNAs in pancreas tissue of HFC-fed mice.

The relative mRNA level of each gene (indicated by fold change) was determined by calibrating against its respective chow-fed group at each time point. The blank indicates genes with no signal or low copy. We did not obtain useful RNA samples from lymph nodes at 26 weeks and from pancreas at 6 weeks.
